# Neural Correlates of Semantic Inhibition in Relation to Hypomanic Traits: An fMRI Study

**DOI:** 10.3389/fpsyt.2018.00108

**Published:** 2018-04-04

**Authors:** Delphine Raucher-Chéné, Sarah Terrien, Fabien Gierski, Alexandre Obert, Stéphanie Caillies, Chrystel Besche-Richard, Arthur Kaladjian

**Affiliations:** ^1^Psychiatry Department, University Hospital, Reims, France; ^2^Cognition, Health and Socialization Laboratory (EA 6291), University of Reims Champagne-Ardenne, Reims, France; ^3^Speech and Language Laboratory (UMR 7309), CNRS, Aix-en-Provence, France

**Keywords:** hypomanic personality, fMRI, mania, semantics, vulnerability

## Abstract

**Objectives:**

Language modifications are a core feature of mania, but little is known about the semantic mechanisms behind these disturbances. The aim of the present study was thus to identify deficits in semantic inhibition and their respective neural activation patterns in a sample of individuals assessed for hypomanic personality traits.

**Methods:**

Thirty-six young adults with no neurological or psychiatric diagnoses were assessed for hypomanic personality traits with the Hypomanic Personality Scale (HPS) and underwent an fMRI task of semantic ambiguity resolution.

**Results:**

Regression analyses revealed a positive association between the HPS score and activity in the left superior frontal gyrus, left inferior parietal lobule, and anterior cingulate gyrus during semantic ambiguity resolution.

**Conclusion:**

We found a link between HPS scores and brain areas that are part of the cognitive control loop and semantic memory network during language processing in a nonclinical sample of individuals. The hyperactivation of these regions may reflect a compensatory neural response in a population with greater vulnerability to BD.

## Introduction

Clinical features of mania include elevated mood and grandiosity, and several signs such as hyperactivity, accelerated thought processes, and pressured speech (1, 2). One of the most salient features of manic patients is that they are talkative, with disorganized speech ranging from digression to flights of ideas (2, 3). However, little is known about the cognitive mechanisms behind these disturbances. Some studies have indicated that semantic processing seems to be impaired, but results are discrepant (4, 5). The lack of knowledge about the underlying mechanisms of this mood state may be due to the symptomatology itself, which is hardly compatible with an extensive and thorough cognitive exploration. Within this framework, trait-like tendencies toward manic symptoms may be well worth exploring, as there is evidence for a dimensional structure supporting the idea of an affective spectrum from non-pathological highs through to hypomania and mania (6–8). Hypomanic traits are present in the general population to varying degrees and can be assessed with the Hypomanic Personality Scale (HPS) (9). Individuals with high levels of hypomanic traits are described as cheerful, optimistic, extraverted, self-confident, and energetic, but sometimes also irritable, rude, and reckless or irresponsible (10). These personality traits have been associated with positive aspects, the so-called “bright side” of hypomania, as higher mental toughness, ambition related to achieving social recognition, greater creativity or romantic love intensity (11–14). Nevertheless, the presence of high levels of hypomanic traits has also been linked to “dark side” hypomania symptoms or cognitive impairments (15). For instance, participants with high hypomanic traits have been found to be impaired on emotion processing and impulsivity tasks (16–19). Individuals with high levels of hypomanic traits are also too talkative, think faster, or may make more jokes or puns (7, 9). But relatively little is known about the underlying cognitive mechanisms of language or thought disorders in hypomania. To the best of our knowledge, only two studies have so far investigated the relationship between language processing and hypomanic traits (20, 21). Both were conducted by our team and used event-related potentials (ERPs) to assess the electrophysiological component of language processing. The first one investigated the integration of contextual information in emotional situations, showing that participants with higher hypomanic traits exhibit specific modulation of the N400 component (20). The second one investigated the ability to inhibit semantic content in relation to HPS scores (21), with participants performing a semantic ambiguity resolution task adapted from Hoenig and Scheef (22). In this task, the successful disambiguation of homophony putatively requires semantic activation of the possibly relevant meaning and cognitive control over the contextually irrelevant meaning through semantic inhibition (23). Results revealed a positive relationship between scores on the HPS Social Vitality subscale and modulation of the N400 component in the frontal region of interest in the incongruent unambiguous (IU) condition and in the frontocentral region of interest in the incongruent ambiguous (IA) condition (21). The results of this study therefore suggest that individuals with high levels of hypomanic traits have difficulty handling the competition between different meanings of ambiguous words. However, the source of these specific ERP patterns could not be located, owing to the limited number of electrodes used.

Accordingly, the aim of the present study was to further explore individuals’ ability to inhibit semantic content in relation to their hypomanic traits and to identify the underlying neural activation patterns. In the general population, the neural correlates of semantic ambiguity processes have been found in the frontotemporoparietal cortex and subcortical areas (24, 25). The activation likelihood estimation meta-analysis conducted by Rodd et al. (25) indicated that the left inferior frontal gyrus (IFG) and superior temporal gyrus play a critical role in semantic processing, as do cognitive control network areas such as the dorsolateral prefrontal cortex and inferior parietal (IPL) cortex in semantic inhibition (22). Based on these previous studies of semantic inhibition, we predicted that the higher the HPS score is, the more difficult participants would find it to resolve the ambiguity in the semantic inhibition condition. This difficulty would be reflected by longer reaction times at the behavioral level and by hyperactivation of the neural network underlying the semantic and cognitive control networks (left IFG and superior temporal gyrus; dorsolateral prefrontal cortex and IPL cortex).

## Materials and Methods

### Participants

Participants were recruited either *via* the Internet or by means of advertisements displayed in Reims University Hospital and on the campus of Reims Champagne-Ardenne University. A total of 331 participants agreed to fill out an online HPS (mean score = 18.29 ± 9.06, median score = 18.09). Ninety-six participants accepted to be contacted afterward. From these participants, 36 nonclinical participants were selected. These participants were selected in accordance with the following criteria: they had to be native French speaker, right-handed (as assessed by the Edinburgh Inventory) (26), had normal or corrected-to-normal vision and hearing, and health and safety regulations regarding the use of MRI. Finally, they were pseudo-randomly selected with an equal gender repartition (18 women) as gender may influence neurocognitive functioning and in order to ensure a linear distribution of scores to allow for a dimensional approach (see Figure S1 in Supplementary Material for participants’ scores and subscores). Exclusion criteria included a personal or history of BD, schizophrenia or schizoaffective disorder or current depression according to the DSM-IV-TR (27), recent alcohol and/or drug abuse or dependence, and significant general medical illness (including neurological disorders or head trauma). Demographic data were collected, and verbal intelligence was estimated using the Mill Hill Vocabulary scale that has consistent test–retest reliabilities in excess of 0.90 for several normal adult populations (28, 29). Participants’ past and present psychiatric history was explored by means of the Mini International Neuropsychiatric Interview (30). Hypomanic traits were assessed with the HPS, a 48-item self-report questionnaire (9). The validation of the French-language version of this scale showed good internal consistency (Cronbach’s alpha = 0.90) and a 3-week test–retest reliability (Pearson’s correlation = 0.82) and confirmed the three-factor structure of the scale, with Mood Volatility, Excitement, and Social Vitality subscales (31). The Mood Volatility subscale explores negative, unpredictable mood states, and hypomanic cognition, the Social Vitality subscale gages social potency and vivaciousness, and the Excitement subscale probes the energetic and extremely cheerful mood exhibited by such individuals (32). These three dimensions have been shown to be relatively independent, with Pearson’s correlation coefficients ranging from 0.35 to 0.52 (32). Depressive symptoms were assessed with the Hamilton Rating Scale for Depression (HAM-D) (33). This scale has good internal consistency (standardized coefficient alpha = 0.82) with an appropriate mean inter-item correlation of 0.23 (34). Reliability coefficients for the last two questionnaires on our sample were satisfactory (HAM-D: nonparametric Cronbach’s alpha coefficient = 0.92; HPS: KR-20 = 0.90). The sample’s demographic and clinical characteristics are set out in Table 1.

**Table 1:** Characteristics of the group of participants.

	Mean	SD	Range
Age (years)	27.17	9.77	19–54
Education (years)	13.58	1.84	11–20
Mill Hill score	33.61	3.95	23–41
HAM-D score	1.38	2.03	0–8
HPS total score	18.39	9.40	2–32
Social Vitality subscore	7.92	4.04	1–16
Mood Volatility subscore	7.86	4.22	1–16
Excitement subscore	2.61	2.14	0–6

*HAM-D, Hamilton Depression Rating Scale; HPS, Hypomanic Personality Scale*.

All participants gave their written informed consent before taking part. The study was approved by the regional ethics committee (CCP Est-3, French National Regulatory Authority) and carried out in accordance with the Declaration of Helsinki and its subsequent amendments.

### Semantic Ambiguity Resolution Task

The semantic ambiguity resolution task, derived from the task developed by Hoenig and Scheef (22), featured 90 auditory context sentences, each followed by a written target word. The material has already been described in detail elsewhere (21). Briefly, half the priming sentences ended with a homophone, and the context of the sentence pointed toward the homophone’s subordinate meaning (i.e., ambiguous condition), while the other half ended with a control word that was synonymous with the subordinate meaning of the homophone (i.e., unambiguous condition; see Table 2 for an example). The target word, displayed on a screen, was oriented to either the dominant or the subordinate meaning of the homophone, thus creating incongruent and congruent conditions. This resulted in a total of four conditions: congruent ambiguous (CA), IA, congruent unambiguous (CU), and IU.

**Table 2:** Example of experimental material.

Condition	Heard sentence	Target
Congruent ambiguous (CA)	*Elle danse pour un ballet/*[balε](she’s dancing in a ballet)	*GALA*(gala ball)
Congruent unambiguous (CU)	*Elle danse pour un spectacle*(she’s dancing in a show)	*GALA*(gala ball)
Incongruent ambiguous (IA)	*Elle danse pour un ballet/*[balε](she’s dancing in a ballet)	*MENAGE*(housework)
Incongruent unambiguous (IU)	*Elle danse pour un spectacle*(she’s dancing in a show)	*MENAGE*(housework)

*The homophone [balε] has two meanings: the dominant meaning is broom and the subordinate meaning is dance performance*.

*Underlined words at the end of the sentences are the homophones or the control word*.

**Figure 1:**
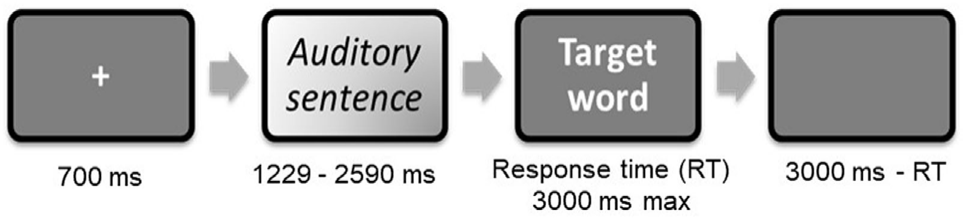
Trial procedure extracted from Raucher-Chéné et al (21).

The stimuli were presented in a fixed, pseudorandom order, using E-Prime 2.0 software (Psychology Software Tools, Pittsburgh, PA, USA). Each trial began with a fixation cross (700 ms), followed by an auditory context sentence (1,229–2,590 ms), and ended with a written target word. This word, printed in white letters on a black background, was displayed for a maximum of 3,000 ms or until the participant pressed a response button, in which case it was followed by a blank screen for the remaining time (i.e., 3,000 ms minus reaction time) (see Figure 1). To optimize the detection of the blood-oxygen-level-dependent (BOLD) signal, jitters were also introduced between successive stimuli. Participants were asked to decide whether the target word on the screen was related to the meaning of the context sentence they had just heard. Half the stimuli required a true (“yes”) response. Two lists of sentences were randomly used, with 22 or 23 sentences per condition, associating the target words with the ambiguous or nonambiguous sentences.

The priming sentences were played through headphones dedicated to fMRI studies, and each target word was projected onto a translucent screen (28 in. wide and 37 in. high) by an Epson EB-G5300 video projector (Epson France, Seiko Epson Corporation). It could be viewed through a prismatic mirror mounted on the head coil.

### Functional MRI Acquisitions

Images were acquired using a 3-T whole-body MRI scanner (Achieva, Philips Medical Systems, Best, The Netherlands) with an eight-channel head coil. Head motions were minimized with a forehead strap and comfortable padding around the participant’s head. For each participant, a *T*1-weighted anatomical image oriented parallel to the AC–PC was first acquired using a fast-field echo sequence (*T*1-FFE; TR = 253 ms, TE = 2.30 ms, flip angle = 80°, 38 axial slices, slice thickness = 4.50 mm, no interslice gap, FOV = 240 mm × 240 mm, matrix = 268 × 214, and acquisition voxel size = 0.43 mm × 0.43 mm × 4.5 mm). Parameters of acquisition were the same as in Carre et al. (35) conducted on the same machine. Functional data were acquired using an ascending slice acquisition 2D-*T*2*-weighted EPI sequence sensitive to BOLD contrast, in the same axial plane as the *T*1-weighted structural images (2D-*T*2*-FFE-EPI; EPI factor = 39, TR = 2,000 ms, TE = 30 ms, flip angle = 90°, 38 axial slices, slice thickness = 3 mm, no gap, matrix = 80 × 72, FOV = 240 mm × 216 mm, acquisition voxel size = 3 mm × 3 mm × 4.5 mm). The 560 functional volumes were collected during two consecutive functional sessions (total scan time = 18 min and 40 s).

### Image Processing and Statistical Analysis

One participant’s data were excluded from analysis, owing to technical issues during the recording of the fMRI session. Image processing and statistical analyses were conducted using statistical parametric mapping methods as implemented in SPM12 (http://www.fil.ion.ucl.ac.uk/spm/software/spm12/). Functional images were slice-time corrected and spatially realigned to the first volume. Structural images were then co-registered to the mean realigned EPI image and segmented. These segmentation parameters were used to normalize functional data to the standard anatomical space of the Montreal Neurological Institute brain. Functional images were resampled at a resolution of 2 mm × 2 mm × 2 mm. Spatial smoothing was performed with an isotropic three-dimensional Gaussian filter with a full width at half maximum of 8 mm. A high-pass filter was implemented using a cutoff period of 128 s to remove low-frequency drift from the time series.

At the first level, a design matrix was defined with a separate regressor for each condition (CA, IA, CU, and IU), and contextual sentences were entered as an additional regressor. Motion parameters extracted from the realignment processing were also included in the model. From this first-level model, four contrasts were computed for each of the four experimental conditions.

The resulting images of the first-level analyses were entered into a second level, with a flexible factorial design. This model included two two-level factors: congruence (congruent vs. incongruent) and ambiguity (ambiguous vs. unambiguous), in accordance with the analyses of Hoenig and Scheef (22). From this model, we computed the positive main effects of congruence (IA vs. CA and IU vs. CU) and ambiguity (IA vs. IU and CA vs. CU). To explore modulations in activation owing to hypomanic traits when resolving ambiguity in the semantic inhibition condition, we also computed regression matrix in SPM onto the IA > IU contrasts (i.e., semantic ambiguity resolution in incongruent conditions) from the first-level analyses and including the HPS scores as predictors. HAM-D score was added as a covariable in the multiple regression matrix. In line with the previous fMRI study on this task (22) and the recommendations by Woo et al. (36), results were thresholded at *k* = 20 contiguous voxels and *p* < 0.001 uncorrected. No activation remained significant after FWE correction for multiple comparisons at *p* < 0.05 across the whole brain.

Demographic and task performance measures were analyzed using IBM SPSS Statistics Version 20.0 (IBM Corp., Armonk, NY, USA).

## Results

### Behavioral Performance During fMRI

The percentage of correct responses was >80% in all conditions. The mean reaction times analyzed using an analysis of variance showed a significant effect of congruity, *F*(1, 34) = 21.53, *p* < 0.001, with shorter reaction times in congruent conditions (incongruent: 1,028.80 ms vs. congruent: 907.21 ms) and a significant effect of ambiguity, *F*(1, 34) = 6.79, *p* = 0.014, with longer reaction times in ambiguous conditions (unambiguous: 954.98 ms vs. ambiguous: 981.03 ms).

We also calculated correlations between HPS scores and both the mean number of correct responses and the mean reaction time for each condition. No significant correlations were observed (*p*s > 0.29), even when controlled by HAM-D scores (*ps* > 0.13).

### Functional MRI Results

**Figure 2:**
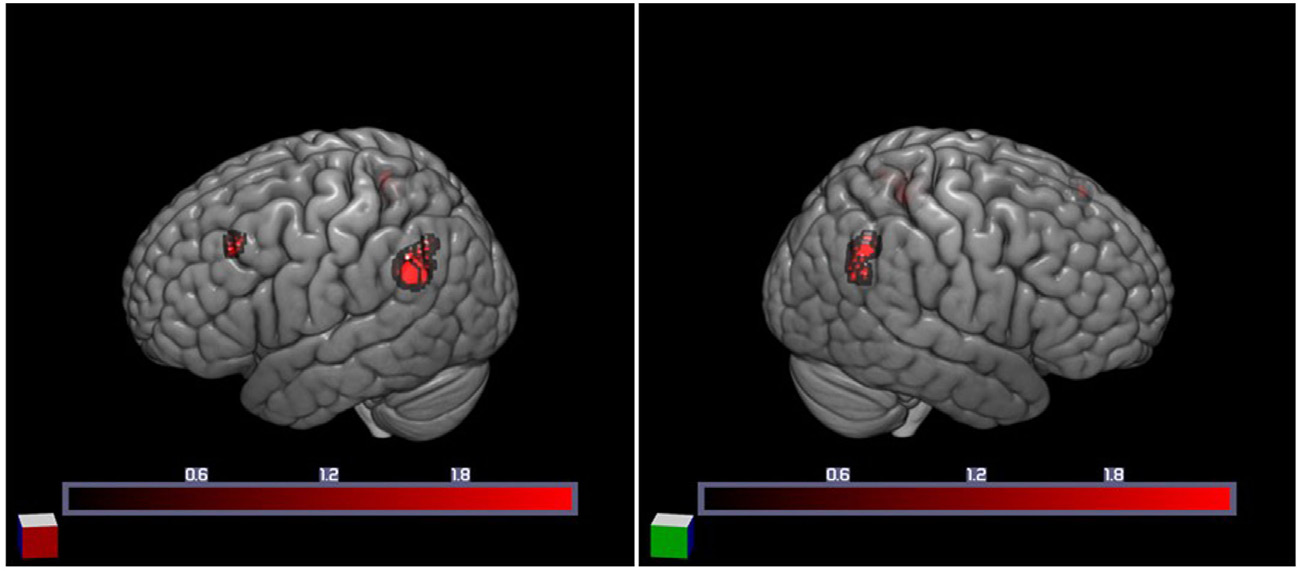
Increased cortical activation for semantic ambiguity contrasted with unambiguity. Activation in the bilateral angular gyri (BA 39) and left middle frontal gyrus (BA 9), as projected on the cortical surface (left and right).

We first examined individual differences in the activation of brain regions involved in the semantic processing of ambiguity and congruity. A positive main effect of incongruity was found in a distributed bilateral network within frontal and parietal brain areas, associated with left temporal and subcortical (basal ganglia) activation (Table 3). A positive main effect of target-related ambiguity processing was found in the bilateral angular gyri (AG) and left middle frontal gyrus (Table 4; Figure 2). These main effects were not modulated by interactions between ambiguity and congruity.

**Table 3:** Semantic incongruity processing: maxima of activation clusters showing a positive main effect for targets in the incongruent conditions compared with targets in the congruent conditions.

Area	BA	Significance			Coordinates		
		Cluster size	*t*-value	*p*_FWE_	*x*	*y*	*z*
L angular gyrus	40	1,188	6.94	0.001	−32	−52	42
L precentral gyrus	44	1,712	6.60	0.001	−46	8	32
R SMA	6	573	5.56	0.001	6	12	52
R IFG triangularis	45	894	5.49	0.001	52	24	26
L precuneus	7	302	4.89	0.012	−6	−70	38
R angular gyrus	7	367	4.71	0.005	34	−54	46
L insula	20	87	4.59	0.338	−30	28	0
L thalamus	–	206	4.42	0.047	−8	−12	4
R precentral	44	69	4.32	0.458	36	6	32
R pallidum	–	58	4.17	0.548	18	10	2
L putamen	–	95	4.16	0.295	−12	10	4
L MTG	21	24	3.63	0.868	−48	−44	8
L PCC		36	3.61	0.756	−2	−38	22

*L, left; R, right; SMA, supplementary motor area; IFG, inferior frontal gyrus; PCC, posterior cingulate cortex; MNI, Montreal Neurological Institute; p_FWE_, cluster-corrected threshold for familywise error*.

*p < 0.001, uncorrected*.

*k = 20*.

**Table 4:** Semantic ambiguity processing: maxima of activation clusters showing a positive main effect for targets in the ambiguous conditions compared with targets in the unambiguous conditions.

Area	BA	Significance			Coordinates		
		Cluster size	*t*-value	*p*_FWE_	*x*	*y*	*z*
L angular	39	295	4.62	0.013	−54	−54	36
L MFG	9	23	3.87	0.877	−36	28	46
R angular	39	138	3.79	0.142	50	−56	40

*L, left; R, right; MFG, middle frontal gyrus; p_FWE_, cluster-corrected threshold for familywise error*.

*p < 0.001, uncorrected*.

*k = 20*.

**Figure 3:**
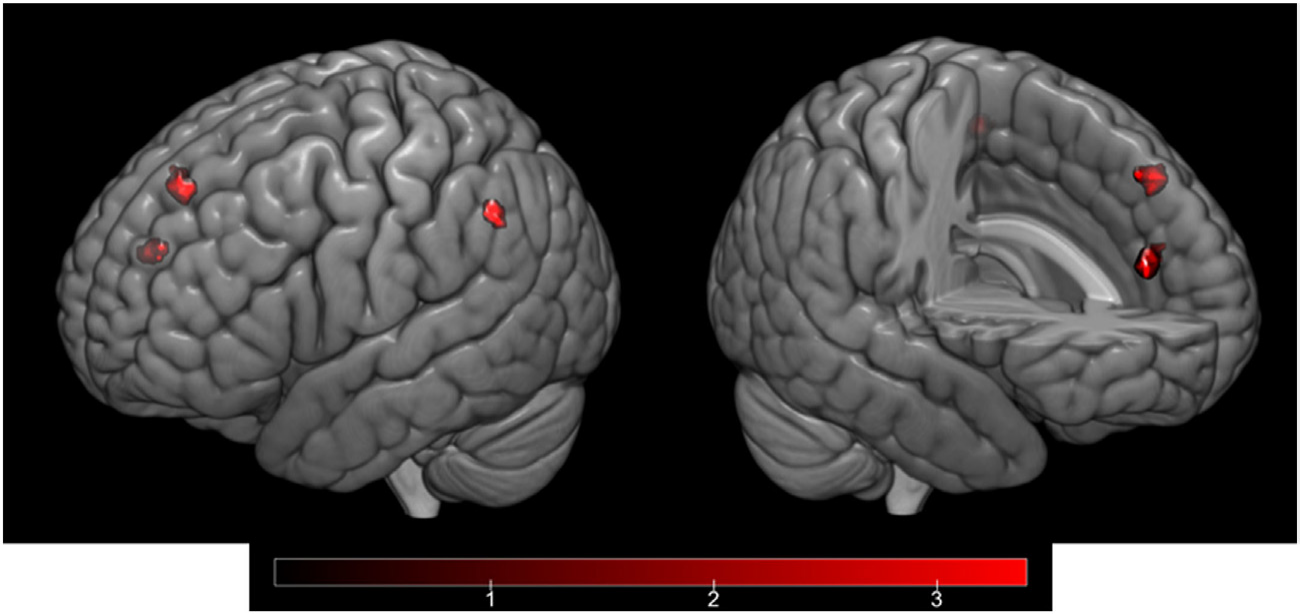
Cortical activation corresponding to the regression analysis of the IA > IU contrast and the Hypomanic Personality Scale (HPS) Social Vitality subscore, controlled by Hamilton Rating Scale for Depression (HAM-D) scores and inclusively masked by the IA > IU contrast. *Activation in the left medial superior frontal gyrus (BA 8), left inferior parietal gyrus (BA 40), and right anterior cingulate gyrus (BA 32) as projected on the cortical surface (left) and on a sagittal section (right)*.

Ambiguity resolution (IA > IU contrast) resulted in significant activation of the left AG (Brodmann area, BA 39). Moreover, a regression analysis between the ambiguity resolution contrast and HPS total score revealed activation of the left medial superior frontal gyrus (SFG) (BA 9) and left IPL gyrus (BA 40). Regarding the HPS subscores, a regression analysis on the Social Vitality subscore highlighted activation in the left medial SFG (BA 8), left IPL gyrus (BA 40), and right anterior cingulate gyrus (ACG) (BA 32) (Table 5; Figure 3). No significant activation was revealed by regression analyses on the Mood Volatility and Excitement subscores.

**Table 5:** Regression analysis of IA > IU contrast and Hypomanic Personality Scale (HPS) scores, controlled by Hamilton Rating Scale for Depression (HAM-D) scores and inclusively masked by the IA > IU contrast.

Area	BA	Significance			Coordinates		
		Cluster size	*t*-value	*p*_FWE_	*x*	*y*	*z*
**IA > IU and HPS total**							
L medial SFG	9	43	4.50	0.670	−6	38	48
L IPL	40	36	4.37	0.747	−56	−50	42
**IA > IU and HPS social vitality**							
L medial SFG	8	52	4.25	0.573	−8	40	46
L IPL	40	23	4.20	0.882	−56	−50	42
R ACG	32	40	3.86	0.702	4	46	22

*BA, Brodmann area; R, right; L, left; SFG, superior frontal gyrus; IPL, inferior parietal gyrus; ACG, anterior cingulate gyrus; p_FWE_, cluster-corrected threshold for familywise error*.

*p < 0.001, uncorrected*.

*k = 20*.

## Discussion

The aim of the present study was to investigate the modulation of semantic disambiguation and its neural correlates in relation to hypomanic personality traits in a selected nonclinical sample. We expected to observe specific patterns of activation according to the intensity of the hypomanic traits.

First of all, for all participants, results showed the activation of a bilateral network within frontal and parietal cortex areas, associated with left temporal and subcortical activation. These results were in line with previous research on semantic ambiguity resolution, involving semantic and cognitive control networks (22, 25, 37).

Our results revealed a positive association between self-rated hypomanic traits and activation of the left SFG. This structure forms part of the dorsomedial prefrontal cortex (DMPFC), which has been a focus of attention in research on emotion processes, social cognition, self-referential processing, and the default mode (38). The SFG lies between the ventromedial prefrontal areas involved in emotion and reward and the lateral prefrontal networks involved in cognitive control and may act as an intermediary between these processing systems (38). The link between DMPFC activation and the HPS score suggests that the higher the HPS score, the greater the activation of the DMPFC during semantic ambiguity resolution. Callicott et al. (39) suggested that prefrontal regions may initially respond to difficult task demands with an increased level of activation and preserved performances, but that when a ceiling level is reached, the neural response decreases and performances decline. This hypothesis was supported by our results, as the level of activation in the left SFG was positively correlated with the HPS total score, but performance on the semantic ambiguity task was preserved, regardless of the HPS score, suggesting that the ceiling was not reached. Our results further suggest that task resolution was more difficult for higher HPS scorers, although no differences were found at the behavioral level.

The left IPL lobule was also more activated in individuals with higher HPS scores. Functional imaging studies in healthy populations have suggested that this region is specifically involved in representational aspects of semantic memory. However, AG activation has also been observed in anticipatory attentional control (40) and conflict resolution processes (41). Compared with the right AG, the activation of the left AG may reflect a strong contextual/semantic conflict. In BD, the reduced engagement of the cognitive control network including the dorsal anterior cingulate cortex, dorsolateral prefrontal cortex, ventrolateral prefrontal cortex, and IPL cortex may lead to reduced inhibition (42).

Regarding the HPS subscores, activation related to the Vitality subscore was comparable to that observed for the HPS total score, with additional activation of the ACG. The ACG is known to be involved in the detection and evaluation of errors and/or conflict (43) and interacts with the dorsolateral prefrontal cortex to form a cognitive control loop (44). In a recent meta-analysis of cognitive control tasks across DSM Axis I disorders, McTeague et al. (45) observed a transdiagnostic pattern of aberrant brain activation in regions corresponding to the well-established multiple-demand network, including the left prefrontal cortex (premotor to mid-dorsolateral prefrontal cortex) and anterior mid-cingulate cortex. These findings of increased activity in relation to hypomanic trait intensity can be conceptualized as a compensatory neural response designed to overcome potential deficits in attentional control and/or increased distractibility. The Social Vitality subscale estimates social potency and vivaciousness, but also reflects impulsivity traits (32). In our previous work conducted with ERPs on the same task, we found that the difficulty participants with higher HPS scores had handling the competition between different meanings of ambiguous words was related to modulation of the amplitude of the N400 component in frontal and frontocentral areas, and this modulation was in turn correlated with the HPS Social Vitality subscore (21). Taken together, these results provide evidence that high levels of hypomanic personality traits are associated with specificities in the processing of ambiguity resolution at the electrophysiological and neuroanatomical levels. As trait-like tendencies toward manic symptoms seem to be part of a continuum from normal to pathological mood states (8), our results may also improve current understanding of the language disturbances observed in BD. The possible predictive power of hypomanic traits and their attributes have been discussed in prospective studies showing that 25% of the participants with the higher HPS scores developed a mood disorder of the bipolar spectrum (46, 47). So if we extrapolate our results, we might suggest that the language disorders like pressured speech and tangentiality that are core features of BD (48, 49) may result from a semantic inhibition impairment specific to the disorder.

Several aspects of the present study limit the generalization of our findings. First, hypomanic traits were assessed on the HPS, which is a self-report questionnaire, and some participants may have over- or underestimated their levels of hypomania. Second, it should be borne in mind that vulnerability markers are differently distributed among patients vs. healthy controls, more prevalent among family members, associated with spectrum disorders in family members, and present before the manifestation of clinical symptoms. Moreover, their reliability and stability increase over time (50). Our study therefore paves the way for further explorations, which should focus on patients with BD presenting mild levels of hypomania as well as their unaffected relatives to confirm our results.

## Ethics Statement

All participants gave their written informed consent before taking part. The study was approved by the regional ethics committee (CCP Est-3, French National Regulatory Authority) and carried out in accordance with the Declaration of Helsinki and its subsequent amendments.

## Author Contributions

DR-C, ST, FG, SC, CB-R, and AK participated in the conception and design of the study; DR-C, ST, FG, and AO conducted the data acquisition and analysis; DR-C, ST, FG, and AO contributed to the drafting of the manuscript; and SC, CB-R, and AK revised it critically. All the authors read and approved the final version.

## Conflict of Interest Statement

The authors declare that the research was conducted in the absence of any commercial or financial relationships that could be construed as a potential conflict of interest.

## Supplementary Material

Figure S1 HPS score and sub-scores for each participant (n = 36).
