# Supplementary figures and images for: Neural Correlates of Semantic Inhibition in Relation to Hypomanic Traits: An fMRI Study
Source: Front Psychiatry. 2018 Apr 4;9:108. doi: 10.3389/fpsyt.2018.00108 (PMC5893717; doi:10.3389/fpsyt.2018.00108)

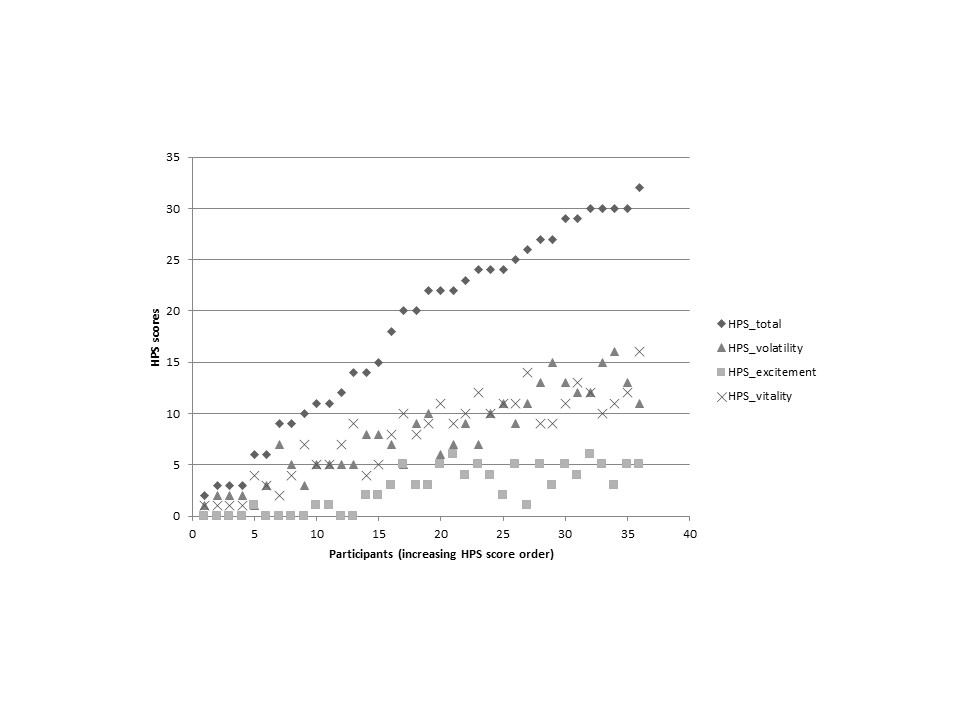

Supplement: Figure S1 — HPS score and sub-scores for each participant (n = 36). [file Image_1.jpeg]
